# Flow cytometry study of *Escherichia coli* treated with plasma-activated water: confirming the absence of the viable but non-culturable state in bacteria

**DOI:** 10.3389/fmicb.2025.1592471

**Published:** 2025-06-04

**Authors:** Rita Agus, Fabio Avino, Aleksandra Lavrikova, Brayden Myers, Ivo Furno

**Affiliations:** Department of Physics, Swiss Plasma Center (SPC), École Polytechnique Fédérale de Lausanne (EPFL), Lausanne, Switzerland

**Keywords:** Plasma-activated water (PAW), low-temperature plasmas (LTPs), flow cytometry, live/dead BacLight PI/SYTO9 staining, viable but non-culturable bacteria

## Abstract

Plasma-activated water (PAW) is an emerging antimicrobial agent with promising applications in bacterial inactivation. The PAW samples are generated by non-contact exposure of deionized water to a surface dielectric barrier discharge plasma and are characterized by a reactive nitrogen species-rich chemistry. In this work, fluorescence flow cytometry is applied to assess the viability of *Escherichia coli* treated with PAW. The results indicate that PAW exhibits a strong bactericidal effect, significantly increasing propidium iodide positive populations and leading to cell shrinkage. Comparative colony-forming unit counting confirmed these findings, showing agreement between both techniques and ruling out the possibility of a viable but non-culturable (VBNC) bacteria state. These results underscore the potential of the PAW samples produced with the present setup for safe decontamination applications, while also offering insights into the mechanisms of bacterial inactivation.

## 1 Introduction

Plasma-activated water (PAW) is a novel and promising technology in the decontamination sector, offering an alternative to traditional chemical and heat-based disinfectants (Wang and Salvi, 2021). PAW is produced by exposing water to low-temperature plasmas (LTPs), leading to the accumulation of reactive oxygen and nitrogen species (RONS) within the water (e.g., O_3_, H_2_O_2_, ${\text{NO}}_{x}^{-}$). Exposure to LTPs also alters pH, electrical conductivity (EC), and oxidation reduction potential (ORP), inducing water acidification and an increase in EC and ORP (Tian et al., 2015; Zhou et al., 2018; Hadinoto et al., 2021). The synergistic effect of RONS and low pH is believed to be responsible for the antimicrobial effects of PAW, inactivating a broad spectrum of microorganisms, including bacteria, viruses, and fungi (Zhou et al., 2018, 2020).

Typically, the efficacy of antimicrobial agents is evaluated by measuring bacterial viability based on their proliferation. Various methods for bacterial counting have been developed, categorized into bulk methods or single-cell measurements. Bulk methods, which are relatively simple and fast, detect microbial growth by measuring changes in turbidity, gas pressure, or conductivity of the liquid medium (Nebe-Von-Caron et al., 2000). Alternatively, they rely on counting bacterial colonies on agar plates, assuming that each colony originates from a single microorganism (CFU counting) (Shapiro, 2000; Koch, 2007). Bulk methods are constrained by the necessity to cultivate bacteria in a controlled environment and do not take into account bacterial heterogeneity. Furthermore, the comparison between single-cell analysis and bulk methods can present discrepancies, as in cases of bacterial injury, presence of persister or dormant cells, or severe starvation, bacterial growth may become temporarily undetectable (Nebe-Von-Caron et al., 2000). The presence of viable but non-culturable (VBNC) cells within the culture might also be the cause of these discrepancies (Fakruddin et al., 2013; Ambriz-Aviña et al., 2014). The VBNC state is a physiological state in which bacteria are metabolically active but cannot duplicate on conventional culture media. Under favorable conditions, VBNC cells may resuscitate and regain their ability to duplicate. The VBNC state is an adaptive survival mechanism employed by many bacterial species, including numerous human pathogens, in response to environmental stressors. Although VBNC bacteria evade detection by conventional culturing methods, they remain viable and can retain virulence potential (Fakruddin et al., 2013; Liu et al., 2023). Environmental stressors such as UV radiation, exposure to toxic compounds, and nutrient deprivation have been shown to induce the VBNC state in bacteria (Trevors, 2011). Therefore, validating a decontamination method requires assessing the presence of the VBNC state. This verification can be achieved by single-cell methods such as flow cytometry (Fleischmann et al., 2021), which, combined with fluorescent staining, is increasingly recognized as a powerful tool for studying bacterial viability (Shapiro, 1985, 2000; Ambriz-Aviña et al., 2014).

A flow cytometer is a device that analyzes single cells by exposing them to a laser beam. The cell under investigation by the diagnostics scatters light in all directions. Forward scattering (FSC), defined as the light scattered at acute angles, is proportional to the particle size, while side scattering (SSC), produced at wide angles, contains information about the internal complexity of the particle (e.g., granularity). In addition to events enumeration, FSC and SSC, fluorescent dye staining allows bacteria species and biological activities to be distinguished, selectively targeting biochemical or structural properties of the cell (e.g., enzyme activities, nucleic acid sequences) (Veal et al., 2000). To select distinct subpopulations of cells within a heterogeneous sample, gating is performed based on the intensity of FSC, SSC, and fluorescence (Shapiro, 1985, 2000; Veal et al., 2000; Ambriz-Aviña et al., 2014).

Assessing bacterial viability with fluorescent flow cytometry is possible through the verification of metabolic activity, maintenance of membrane potential, or membrane impermeability to specific dyes (Shapiro, 2000). Membrane integrity and membrane potential can be measured through dye retention or exclusion methods, often based on multicolour labeling. One of the most common method for assessing membrane integrity is the exclusion of the red-fluorescent nucleic acid stain propidium iodide (PI), combined with the highly membrane-permeable DNA stain, SYTO9. Cells with an intact cytoplasmic membrane are impermeable to charged dyes such as PI. If membrane integrity is lost, PI binds to DNA, increasing the PI fluorescence 20- to 30-fold (Stiefel et al., 2015). Staining with PI is therefore a viability assessment test based on dye exclusion. On the contrary, the green-fluorescent nucleic acid stain SYTO9 permeates both live and dead bacterial cells. The SYTO9 fluorescent signal intensifies significantly upon binding to nucleic acid while displaying low intrinsic fluorescence signal when unbound. In the presence of both dyes, PI demonstrates a greater affinity for nucleic acids than SYTO9, leading to the displacement of SYTO9 by PI (Nebe-Von-Caron et al., 2000; Stiefel et al., 2015).

The live/dead BacLight staining kit (Invitrogen, 2004) utilizes SYTO9 and PI for viability determination and VBNC cell detection. This method has been widely used and extensively validated through microscopy and flow cytometry analyses (Lisle et al., 1998; Auty et al., 2001; Leuko et al., 2004; Berney et al., 2007; Taimur Khan et al., 2010; Ambriz-Aviña et al., 2014). It has been applied for microbial enumeration and detection (Leuko et al., 2004), as well as for antiseptic efficacy testing (Langsrud and Sundheim, 1996; Stocks, 2004). Additionally, its applications extend to water quality assessment (Endo et al., 1997; Boulos et al., 1999) and hygiene monitoring in processed meat (Duffy and Sheridan, 1998). These studies have shown a strong correlation between microscopic counts and direct viable counts, with discrepancies of ~ 0.1 log/mL (Boulos et al., 1999). This difference corresponds to about a 20% variation in percentage of inactivation.

It is important to acknowledge that PAW, as other decontamination methods, may not always induce complete bacterial cell death but could instead cause sublethal damage and resulting in VBNC state. Several studies have reported bacterial VBNC state following plasma treatment (Cooper et al., 2010; Kvam et al., 2012; Xu et al., 2018; Zhao et al., 2020). For instance, Dolezalova and Lukes (2015) observed a nearly 7-log reduction in *E. coli* populations following 15 min of plasma exposure when assessed by CFU counting. However, the same study reported a much smaller reduction (< 1 log, corresponding to 45% of inactivation) when viability was determined using the live/dead staining technique, suggesting that a significant proportion of bacteria had transitioned into the VBNC state (Dolezalova and Lukes, 2015). These findings underscore the need for complementary analytical techniques beyond traditional CFU counting to accurately assess bacterial viability following PAW treatment. Several microscope acquisitions of bacteria treated with PAW and stained with the live/dead BacLight kit, such as *Staphylococcus aureus* (Ma et al., 2015; Tian et al., 2015), *Listeria monocytogenes* (Handorf et al., 2021), and *Escherichia coli* biofilms (Xia et al., 2023), are present in the literature, but they were not associated with quantitative flow cytometry (Gan et al., 2022).

This study aims to address a critical gap in bacterial viability assessment by utilizing fluorescent flow cytometry to investigate the potential induction of the VBNC state in *E. coli* following treatment with PAW. Understanding whether PAW drives *E. coli* into a VBNC state is crucial for accurately evaluating bacterial inactivation beyond conventional culture-based methods and has significant implications for antimicrobial applications. Moreover, this study specifically tests VBNC induction of reactive nitrogen species (RNS)-rich PAW in contrast with existing research that predominantly focuses on PAW chemistries dominated by reactive oxygen species (ROS) (Ma et al., 2015; Gan et al., 2022; Xia et al., 2023).

## 2 Materials and methods

### 2.1 PAW reactor

**Figure 1:**
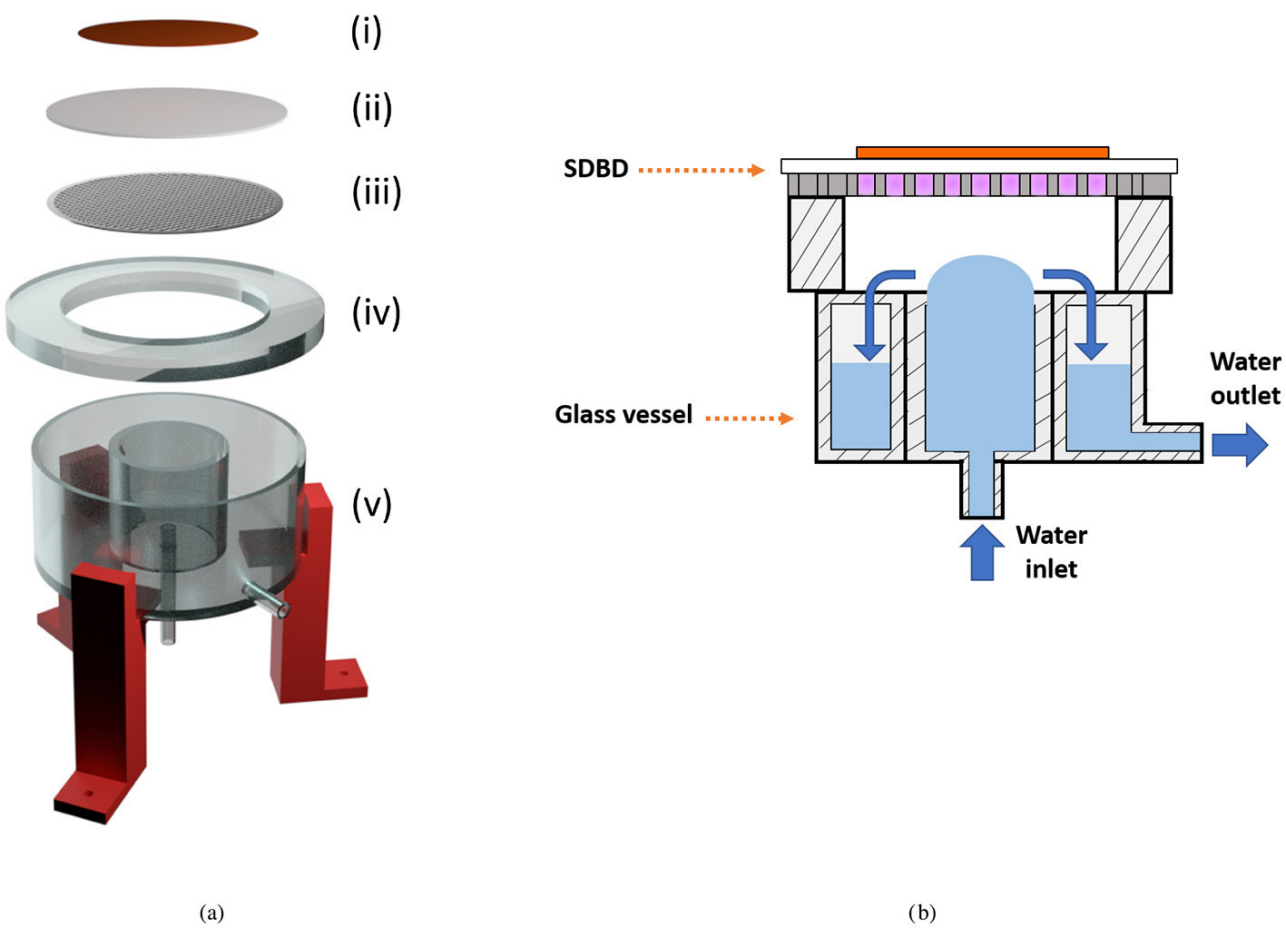
**(a)** Expanded view of the PAW reactor setup. The SDBD consists of a copper back-plate electrode (i), a dielectric disk (ii), and a stainless steel perforated disk electrode (iii). The SDBD is in contact with the glass spacer (iv), located on top of the glass vessel (v). A red 3D-printed structure supports the setup (Agus et al., 2024b). **(b)** Sketch of the assembled PAW reactor, illustrating the SDBD with the plasma on and the water configuration in the glass vessel. Blue arrows indicate the water flow direction.

The reactor used for the preparation of the PAW samples, illustrated in Figure 1, has been thoroughly described in previous publications (Agus et al., 2024a,b). The portable setup features a surface dielectric barrier discharge (SDBD) system, which consists of a copper back-plate electrode, a dielectric disk, and a stainless steel perforated disk electrode, as depicted in Figure 1a. The SDBD is positioned in contact with a glass spacer placed on top of a glass vessel. As shown in Figure 1b, the water and plasma do not come into direct contact, with a gap of ~ 4 mm between them, guaranteeing no changes in water temperature during the plasma exposure. The ultra-pure water can be recirculated within the glass vessel through a persistaltic pump with a constant flow rate of 200 mL/min. The blue arrows in Figure 1b indicate the direction of water flow when the recirculation system is activated. During the PAW reactor operation, 150 mL of ultra-pure water contained in the glass vessel, was exposed to the SDBD. The SDBD high voltage waveform featured a 8.8 kV peak-to-peak wave at 21 kHz, modulated at 100 Hz, resulting in a plasma discharge power of ~ 39 W, measured by Lissajous figures (Peeters and Butterworth, 2018; Agus et al., 2024a). This relatively high plasma discharge power ensured a nitrogen oxides-dominated chemistry, favoring the formation of nitrogen oxides over reactive oxygen species (Agus et al., 2024a).

The samples are labeled as dynamic PAW or static PAW, depending on whether the water recirculation is on or off, respectively. The chemical composition of PAW is influenced by both the presence of water recirculation and the duration of plasma exposure. To specify the plasma treatment duration, samples are labeled as PAW-XX, where XX denotes the exposure time in minutes.

Two PAW samples were used for this investigation: static and dynamic PAW-20. These samples were chemically characterized in previous works (Agus et al., 2024a,b). The chemical properties of the PAW samples applied in this work are summarized in Table 1. The RONS concentrations reported have been measured by vis-spectrophotometry. The chemical analysis was conducted on PAW samples immediately following plasma exposure. The initial sample consisted of ultra-pure water, obtained from a Puranity TU 3UV/UF water dispenser, with a pH of ~ 6, an oxidation-reduction potential (ORP) of 320 mV, and an electrical conductivity (EC) of 0.055 μS cm-1 (Agus et al., 2024a). These chemical properties are representative of PAW throughout the entire bacterial treatment duration, as PAW samples were used for bacteria treatment immediately after plasma exposure and RONS variations during the first 10 min of storage remain within 10% deviations (Agus et al., 2024b). The setup exhibited remarkable bacteria inactivation capabilities, making it a promising candidate for real-world applications such as surface and instrument decontamination.

**Table 1:** Average chemical properties of the PAW samples applied for the flow cytometry study.

**Sample**	**${\text{NO}}_{2}^{-}$[mg/L]**	**${\text{NO}}_{3}^{-}$[mg/L]**	**H_2_O_2_ [mg/L]**	**EC [μS/cm]**	**ORP [mV]**	**pH**
Static PAW-20	16.9	36.6	0.24	335	554	3.18
Dynamic PAW-20	41.1	29.5	0.46	291	553	3.21

Deviations from the reported values are within 10% (Agus et al., 2024a).

### 2.2 Microbiological protocol

To verify the accuracy of the live/dead BacLight staining under the applied protocol, positive and negative controls have been performed (Invitrogen, 2004). Not treated cells represent the negative control since they should exhibit minimal or no effect on cell viability and strong SYTO9 fluorescence, establishing a baseline for comparison. Positive controls are instead used to validate an expected response, such as a strong signal from the dead-staining component of the kit (PI). Cells treated for 1 hour with isopropanol have been used as positive controls. Studies have reported that isopropanol kills bacteria by enhancing the permeability of bacterial cell membranes and disrupting protein function by denaturing them (Stiefel et al., 2015). After isopropanol treatment, cells, although dead and with a deteriorated membrane, retain their structural integrity (Roth et al., 1997; Stiefel et al., 2015). Isopropanol-treated cells should predominantly exhibit PI fluorescence, indicative of cell death. The experiments were performed with exponentially growing *E. coli* cells with an optical density between 0.2 and 0.5; the cell population was adjusted to 1 × 10^6^ CFUmL^−1^ as recommended by the live/dead BacLight staining protocol. To perform the PAW and isopropanol treatments, aliquots of 1 mL of *E. coli* cells were centrifuged at 4,000 rpm for 3 min. For PAW treatment, 960 μL of supernatant was removed, and the pellet was re-suspended in 960 μL of PAW immediately after plasma exposure. The negative control tubes underwent the same centrifugation cycles and were resuspended in fresh LB to assess any potential effect of the protocol. Tubes were incubated at 37 °C and 180 rpm during 10 min of PAW treatment. For the 1-hour-lasting isopropanol treatment, the cell pellet was re-suspended in 300 μL of pH 7 phosphate-buffered saline (PBS), and 100 μL of 70% of isopropanol were added (Invitrogen, 2004). After the treatment time, the tubes were centrifuged, and the PAW and isopropanol were removed. The pellet was then re-suspended in 1 mL of PBS (pH≈7.4) and the cell suspension was immediately stained. To perform the CFU counting, the cell culture was serially diluted in 10-fold and 100 μL were inoculated in Luria-Bertani agar and incubated at 37 °C overnight. The tubes used for bacterial centrifuging and PAW exposure were 1.5 mL microtubes (Thermo Fisher Scientific Inc.; cat. no 3641NK).

The staining mix (SYTO9 + PI) was prepared in a 1:1 ratio (Boulos et al., 1999; Invitrogen, 2004). The cell suspension was filtered into flow cytometry tubes, and 3 μL of the staining mix was added to each tube designated for staining. After mixing, the tubes were incubated for 15 min at room temperature, protected from light. Before flow cytometry measurements, 100 μL of each suspension was diluted in 700 μL of PBS.

All experiments have been performed in three biological replicates.

### 2.3 Flow cytometry settings

Flow cytometry measurements were performed on a LSR Fortessa, 5-laser and 18-detector analyzer flow cytometer, with 488 nm excitation from a blue solid-state laser operating at 100 mW. Red fluorescence (PI) was measured above 610 ± 10 nm, and green fluorescence (SYTO9) was measured at 530 ± 15 nm. For each measurement, at least 20,000 events were analyzed by flow cytometry, with a flow rate set at ~12 μL/min. The bacterial events were discriminated from debris using FSC-area and side scatter SSC-area. Single cells have been selected for analysis by FSC-height and FSC-area. The gates, applied for population discrimination, were set manually based on the negative and positive controls (Manoil and Bouillaguet, 2018). The percentages of PI and SYTO9-positive cells are then defined over the total stained population, calculated as the sum of cells positive to PI and SYTO9. Data were exported and analyzed with FlowJo software (FlowJo for Windows, Tree Star Inc., Ashland, Oregon, U.S.A.).

## 3 Results and discussion

**Figure 2:**
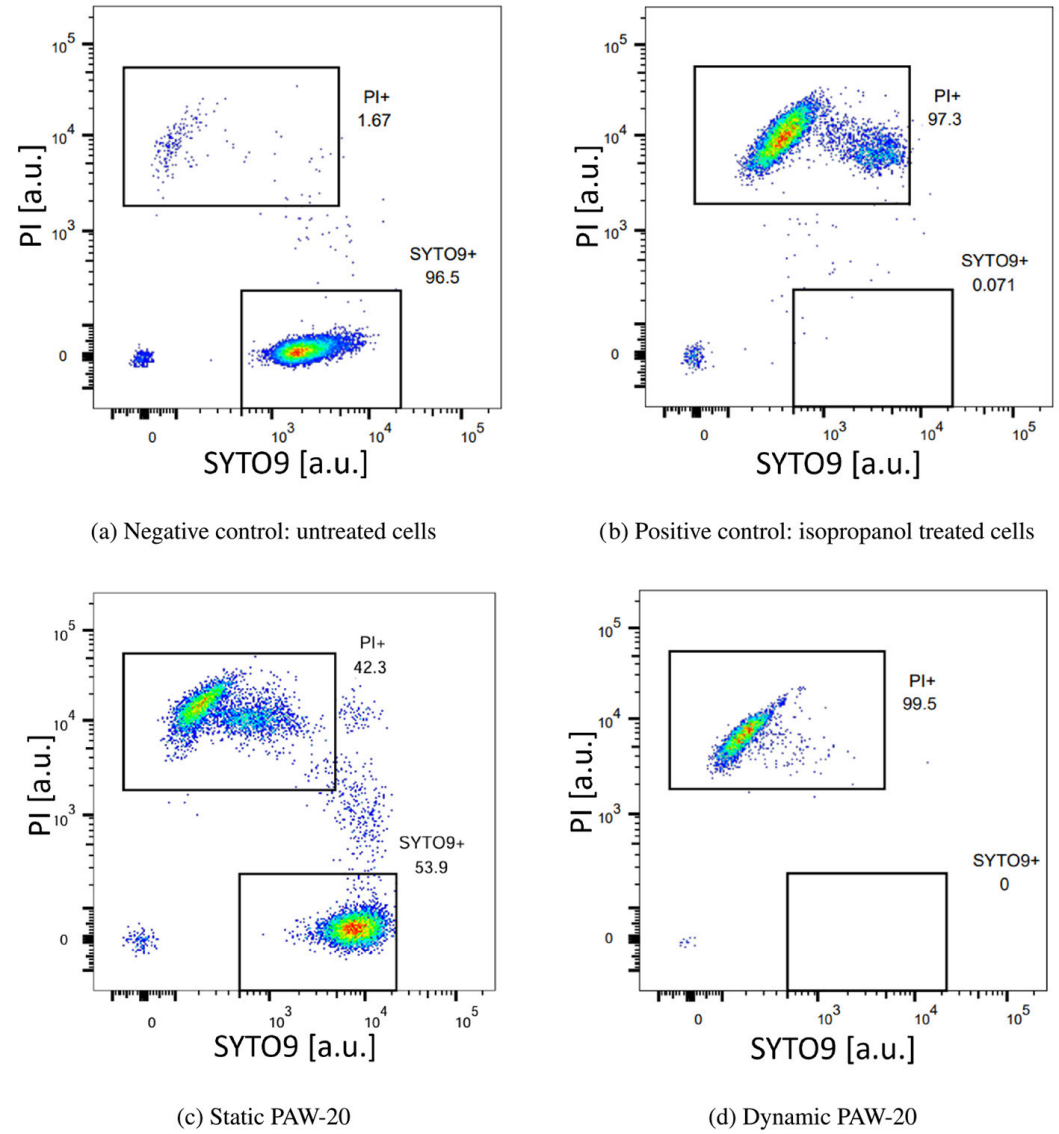
Representative density plots acquired by flow cytometry of *E. coli* negative control **(a)**, positive control treated with isopropanol **(b)**, static PAW-20 **(c)**, and dynamic PAW-20 **(d)** treated *E. coli* stained with a mixture of PI (y-axes) and SYTO9 (x-axes). Plots represent the fluorescence intensity in arbitrary units.

Figure 2 presents four representative flow cytometry graphs displaying PI and SYTO9 fluorescence signal intensities for the negative control (a), positive control (b), and for cells treated with static (c) and dynamic (d) PAW-20. Rectangular gating was applied to distinguish PI-positive (non-viable) cells from SYTO9-positive (viable) cells, with numerical values indicating the percentage of single cells in each category.

In the negative control, the majority of cells were viable and therefore located at the bottom of the graph, exhibiting SYTO9 fluorescence. Conversely, in the positive control (Figure 2b), the cells treated with isopropanol were PI-positive, confirming their loss of viability and clustering on the top part of the graph. The flow cytometry profiles for static and dynamic PAW-20 (Figures 2c, d) revealed a stronger bactericidal effect for dynamic PAW-20, in agreement with previous findings (Agus et al., 2024a). After static PAW-20 treatment, approximately half of the population remained SYTO9-positive, while following dynamic PAW-20 treatment, the majority of cells were categorized as non-viable. Notably, in Figures 2b, c, PI-positive cells appear to segregate into two populations, both with strong PI fluorescence intensities (>10^3^ arb. units). This suggests membrane permeability to PI, confirming non-viability, while still expressing residual SYTO9 fluorescence, indicating that SYTO9 has not been completely quenched by PI. As reported also by Berney et al. (2007), this configuration could be associated with the presence of cells in an unfinished state of division, containing a higher amount of nucleic acid, where part of the SYTO9 fluorescence could still be expressed. Another factor to consider is bacterial heterogeneity since bacteria can exhibit natural variations in physiological characteristics, including size, shape, and DNA content (Berney et al., 2007; Zacharias et al., 2015). Similarly, in Figure 2c, a portion of cells appears to be progressively transitioning from the SYTO9 to the PI gating. The majority of these cells already exhibit relatively high PI fluorescence intensities (of the order of 10^3^ arb. units), indicative of compromised membranes and dying bacteria.

**Figure 3:**
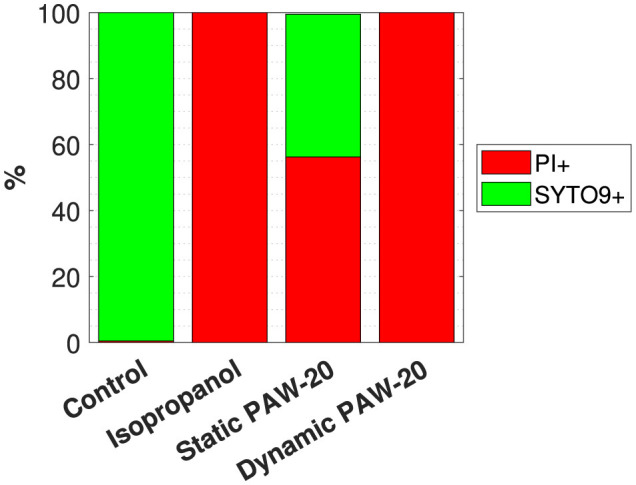
Flow cytometry results indicating the average % of single cells positive to PI and SYTO9 over three biological replicates of the negative control, the positive control treated with isopropanol and static and dynamic PAW-20-treated cells.

The flow cytometry results, averaged over three biological replicates, are reported in Figure 3. The negative control, consisting of untreated bacteria, revealed that on average, 99.9% of single cells were stained with SYTO9. Conversely, in the positive control, where cells were treated with isopropanol, 98.9% of single cells tested positive for PI. Both positive and negative controls delivered the expected results, demonstrating a good performance of the staining. The error bars, not visible in the graph, accounted for a variability of < 1%. Static and dynamic PAW-20 treatments were performed for 10 min. Approximately half of the stained population (56.3%) tested positive for PI after static PAW-20, while dynamic PAW-20 accounted for 99.9% of single cells positive for PI. As shown in Table 1, dynamic PAW-20 exhibited higher ${\text{NO}}_{2}^{-}$ and H_2_O_2_ concentrations, lower ${\text{NO}}_{3}^{-}$ levels, and a similar pH compared to its static counterpart, resulting in enhanced antimicrobial properties and PI permeation.

**Figure 4:**
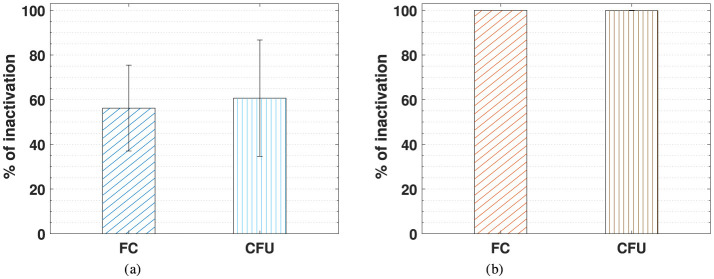
Comparison of the *E. coli* percentage of inactivation measured by flow cytometry (FC) and CFU counting following static PAW-20 **(a)** and dynamic PAW-20 treatment **(b)**.

Figure 4 compares the inactivation percentage of *E. coli* cells treated with static (a) and dynamic (b) PAW-20, assessed by flow cytometry and CFU counting.

The two methods show good agreement, as indicated by the close average values and overlapping error bars. An inactivation of 56.3% was measured by flow cytometry and of 60.7% by CFU counting for static PAW-20. In the case of dynamic PAW the same percentage of inactivation of 99.9% was measured by both flow cytometry and CFU counting. Given the potential for biological variability, the technical challenges inherent in such experiments, and the possibility of instrument error, the discrepancy of about 4% between the two techniques in the case of PAW-20 static is deemed acceptable, especially if considering that the standard deviations associated with CFU counting can be an order of magnitude.

Critically, these findings suggest that bacterial inactivation is not associated with a VBNC state. For both static and dynamic PAW-20, the proportion of inactivated bacteria testing positive for PI closely matched CFU-based inactivation rates, ensuring that PAW treatment achieved reliable and safe bacterial inactivation. Additionally, these results suggest that the presence of PI-positive cells is unlikely to be solely due to temporary permeabilization of the membrane caused by PAW treatment. While such cells would typically stain positive for PI yet retain the ability to recover and proliferate on agar plates, the observed data do not support this as the dominant mechanism. The verification of bacteria inactivation by PAW through flow cytometry and the live/dead BacLight staining performed in this study is of fundamental importance for the validation of these PAW samples as reliable decontamination agents, excluding the possibility of the VBNC bacteria state. For food quality applications, for example, guaranteeing that bacteria are not viable and lack membrane integrity is an important step in safety assessment to avoid potential toxicity arising from metabolite accumulation by non-growing organisms. Metabolic activity and growth depend on the integrity of the cytoplasmic membrane, which shields the cell from its environment. Cells with intact membranes can perform metabolic functions, duplicate, and repair, unless confronted with irreparable DNA damage. Cells lacking an intact membrane are classified as non-viable since they are not capable of maintaining or establishing the negative membrane potential. Their internal structures become exposed to the environment, leading to eventual decomposition (Nebe-Von-Caron et al., 2000).

This flow cytometry study not only quantifies *E. coli* inactivation but also provides valuable insights into the bacterial morphological changes induced by PAW.

**Figure 5:**
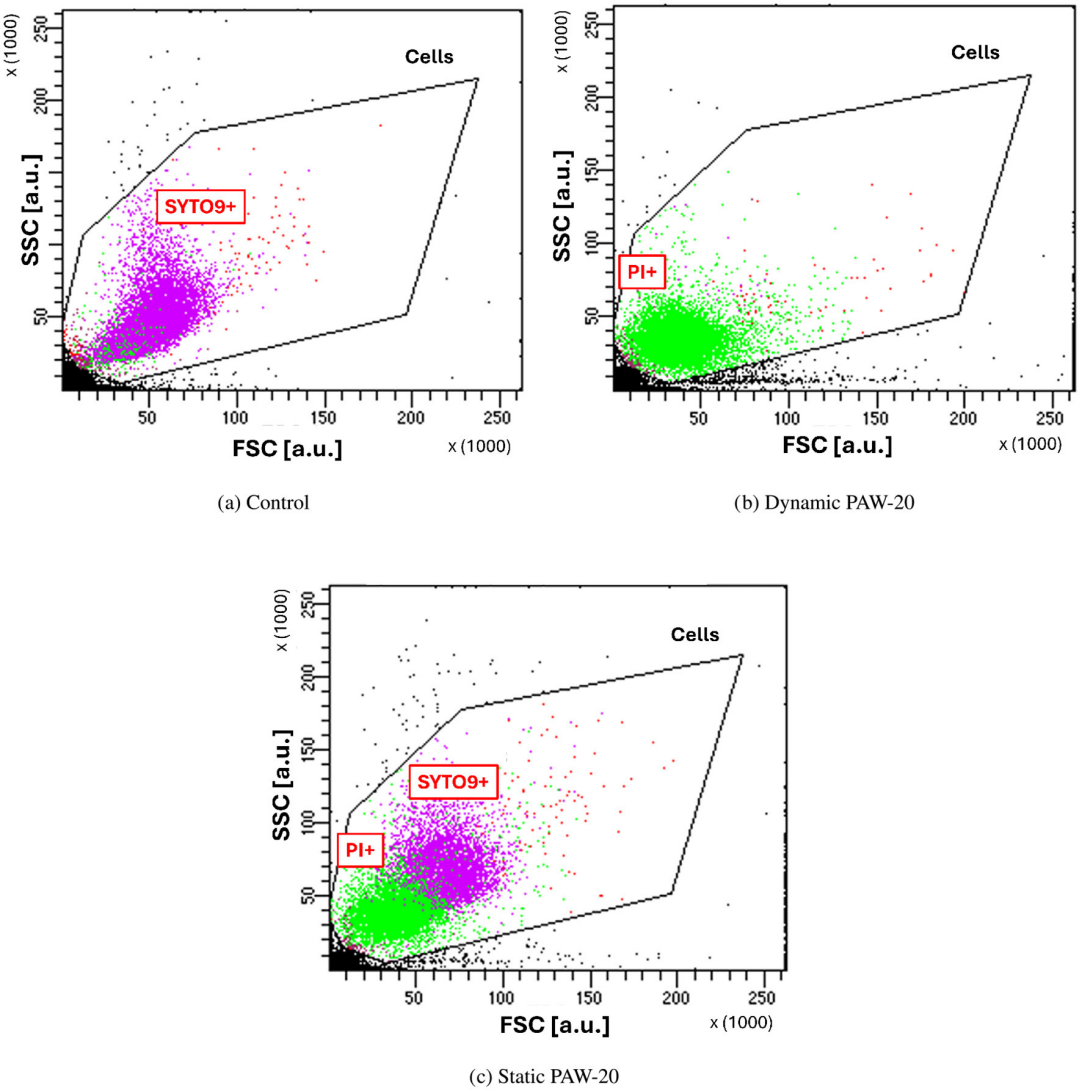
FSC and SSC measured for control cells **(a)**, and dynamic **(b)** and static **(c)** PAW-20-treated *E. coli*. Noticeable cell shrinkage is visible for cells inactivated by the treatment and PI+.

First, the presence of PI-positive bacteria indicates that the bacteria membrane has been compromised or damaged by the treatment (Rosenberg et al., 2019). Possibly, the extent of membrane disruption has to be sufficient to form pores large enough to allow PI molecules to penetrate (≈668.4 Da) (Johnson and Criss, 2013). Second, modifications of the cell size can be observed in the FSC and SSC plots of PAW-treated cells, as demonstrated by Figure 5.

Indeed, FSC intensity correlates with the cell diameter since it is associated with the light diffraction around the cell (FlowJo - Forward Scatter vs. Side Scatter, 2025), while SSC correlates with cell complexity (Vermes et al., 2000; Bortner and Cidlowski, 2007). Control cells, shown in Figure 5a, are almost entirely SYTO9-positive and cluster at higher SSC and FSC values compared to dynamic PAW-treated cells in Figure 5b. Following the sublethal static PAW treatment, a portion of cells remain alive and as shown in Figure 5c, PI-positive and SYTO9-positive populations form two distinct clusters, reflecting differences in cell size. Specifically, SYTO9-positive cells appear larger than PI-positive cells, suggesting that PAW inactivation induced cell shrinkage. The observed cell shrinkage is in agreement with previous findings, where cell shrinkage has been measured by TEM (Gan et al., 2022) and SEM (Zhao et al., 2021). This phenomenon is typically observed in bacteria in association with programmed cell death and membrane blebbing (Bayles, 2014), whereas the opposite, bacterial swelling, is more commonly induced by cell wall-targeting antibiotics, which inhibit cross-linking enzymes and lead to cell lysis (Wong and Amir, 2019).

As previously mentioned, the PAW samples analyzed in this study contain high concentrations of RNS, including nitrite (${\text{NO}}_{2}^{-}$) and nitrate (${\text{NO}}_{3}^{-}$), whereas the hydrogen peroxide (H_2_O_2_) concentrations are two orders of magnitude lower than those of RNS. This suggests that the observed cell shrinkage and membrane damage, detected by flow cytometry, are most likely due to nitrosative stress (Agus, 2025). RNS are well-documented disruptors of essential cellular processes, including protein synthesis and DNA replication, while also compromising membrane integrity and function (Vine, 2011; Chautrand et al., 2022). Peroxynitrous acid is known to be capable of causing membrane poration (Balazinski et al., 2021); however, this hypothesis is excluded here, as both H_2_O_2_ and ${\text{NO}}_{2}^{-}$ are required for its formation (Machala et al., 2018). Since the H_2_O_2_ concentrations are below 0.5 mg L-1 in each tested PAW sample and two orders of magnitude lower than the ${\text{NO}}_{2}^{-}$ concentration, this reactive species is considered negligible (Agus et al., 2024b). Given that the average pH of the PAW samples is below 3.2, the majority of ${\text{NO}}_{2}^{-}$ is expected to be in its protonated form as nitrous acid (HNO_2_), which has a *pK*_*a*_ of 3.3 (Cai et al., 2001). Nitrous acid is known to undergo disproportionation, generating nitrogen oxide (NO), nitrogen dioxide (NO_2_), and nitric acid (HNO_3_) (Rayson et al., 2012; Agus et al., 2024b). In contrast, nearly all of the detected ${\text{NO}}_{3}^{-}$ would remain in its ionic form due to the low acid constant (*pK*_*a*_ = −1.37) (Lukes et al., 2012; Bradu et al., 2020). Notably, NO generated via nitrous acid disproportionation plays a crucial role in post-translational modifications of bacterial membrane proteins, particularly through S-nitrosylation of cysteine residues (Fang and Vázquez-Torres, 2019; Nguyen et al., 2023). At the same time, NO_2_ has been implicated in lipid peroxidation, leading to membrane damage, and it is also capable of oxidizing proteins at multiple sites in a non-specific manner (Halliwell, 1992; Ischiropoulos and Al-Mehdi, 1995; Shuhong, 2016).

## 4 Conclusions

Using florescence flow cytometry, this study demonstrates and quantifies *E. coli* inactivation by PAW generated through the non-contact exposure of ultra-pure deionized water to an SDBD plasma. Flow cytometry analysis confirmed that PAW treatment compromised bacterial membrane integrity, as evidenced by increased propidium iodide uptake in PAW-treated cells.

Comparing the flow cytometry results with CFU counting revealed consistent inactivation rates between the two methods. The agreement between these techniques suggests that PAW effectively inactivates bacteria rather than inducing a viable but non-culturable state, underscoring the reliability of this RNS-rich PAW as a decontamination agent.

Additionally, cell shrinkage was observed, highlighting significant physiological changes induced in *E. coli* by PAW exposure. The predominance of reactive nitrogen species in the PAW used here suggests that nitrosative stress plays a key role in bacterial inactivation, contributing to both membrane damage and cell shrinkage (Agus, 2025). Nitrosative stress is known to disrupt essential cellular processes, including protein synthesis and DNA replication (Vine, 2011; Chautrand et al., 2022).

Overall, these findings, excluding the presence of VBNC cell, reinforce the potential of PAW as an effective and robust bacterial decontamination strategy with promising applications in food safety, healthcare, and industrial sterilization. Other diagnostics, such as energy-dispersive X-ray spectroscopy, v-PCR, and EMA-PCR, could be performed to evaluate bacterial viability and support these findings (Truchado et al., 2020; Haddad et al., 2022; Chen et al., 2022). Additionally, scanning electron microscopy and atomic force microscopy could provide valuable insights into the outer membrane modifications induced by PAW (Eaton et al., 2008).

This study represents an initial step in elucidating the mechanisms underlying *E. coli* inactivation by RNS-rich PAW, suggesting cell shinkage and membrane damage. Future research should focus on molecular-level investigations such as proteomics or RNA sequencing to precisely characterize the pathways involved in PAW-induced bacterial inactivation.

## Data Availability

The raw data supporting the conclusions of this article will be made available by the authors, without undue reservation.
